# Mechanical properties and energy evolution law of marble under the coupled effects of chemical corrosion and dry-wet cycles

**DOI:** 10.1371/journal.pone.0313359

**Published:** 2024-11-18

**Authors:** Yongsheng Liu, Maolin Zhai, Wang Liu

**Affiliations:** 1 School of Civil Engineering and Architecture, East China Jiaotong University, Nanchang, China; 2 Department of Civil Engineering, Nanchang Institute of Technology, Nanchang, China; 3 Department of Management Engineering, Jiangxi Institute of Construction, Nanchang, China; Shenyang Jianzhu University, CHINA

## Abstract

The main factors affecting the safety of underground structures are groundwater chemical corrosion and water level fluctuations. To investigate the mechanical properties of marble and the energy evolution pattern during the failure process under the coupled effects of chemical corrosion and dry-wet cycling, samples were subjected to 5, 10 and 20 cycles of dry-wet ageing in chemical solutions with pH values of 4, 7 and 10, respectively, followed by mechanical property testing. The energy evolution pattern during the failure process of the specimens was also studied. It was found that there is a strong correlation between number of dry-wet cycles and pH value of chemical solution. Chemical corrosion at the early stage of dry-wet cycling has the greatest effect on the deterioration of the rock. As the number of dry-wet cycles increases, the degree of corrosion in acidic solutions is most evident, with the uniaxial compressive strength and elastic modulus decreasing by 27.88% and 33.52% respectively, followed by alkaline solutions, and the degree of corrosion in neutral solutions is the lowest. In addition, dry-wet cycling and chemical corrosion lead to an increase in the internal pores of the rock samples and a decrease in the energy storage capacity. Nevertheless, the proportion of energy loss increases with the number of dry and wet cycles, with the proportion of energy loss in acidic media increasing from 35.61% to 41.63%, indicating that the plastic deformability of marble increases under the action of chemical corrosion and dry and wet cycles. The research results have certain guiding significance for the design, construction and maintenance reinforcement of underground structures under the conditions of chemical corrosion and dry-wet cycling.

## Introduction

Marble, as a common natural material in underground engineering, has physical and mechanical properties that are closely related to the surrounding environment, especially the variation of pH value in the aqueous environment, which significantly affects the mechanical behavior of the rock. Under conditions such as rainfall or fluctuations in groundwater levels, the rock mass may undergo a long-term process of dry-wet cycling. The coupled effects of chemical corrosion and dry-wet cycling inevitably lead to the deterioration of the rock’s physical and mechanical properties, potentially causing safety issues in rock engineering.

In existing research, Lin et al. [1] discovered through uniaxial compressive tests that the porosity of the rock samples increased after chemical corrosion, resulting in a decrease in uniaxial compressive strength and elastic modulus, with the effect of acidic solutions being particularly pronounced. Li et al. [2] demonstrated through uniaxial compressive tests on sandstone subjected to acidic solution immersion that the duration of the consolidation phase in the stress-strain curve was prolonged, and the rock samples exhibited distinct ductile failure characteristics. The performance of rocks under various chemical corrosions, including wet-dry cycles, freeze-thaw cycles, and temperature effects, has been extensively studied, with the mechanical properties exhibiting certain differences [3–6]. Liang et al. [7] analyzed the impact of pH value and corrosion time of chemical solutions on the mechanical properties of sandstone, and consequently established a mechanical damage model. Yu et al. [8] studied the degradation of mechanical properties of limestone under the coupling of chemical solutions and cyclic loading and unloading. Zhou et al. [9] discussed the dynamic mechanical properties and energy evolution laws of granite after chemical corrosion. Huang et al. [10] studied the variation of physical and mechanical properties of different rock materials under high temperature and acidic solution corrosion. Liu et al. [11] conducted a uniaxial compressive test to study the mechanical properties of red sandstone under acid corrosion and proposed a damage constitutive model. Feng et al. [12] examined the damage mechanical properties of deep rocks under high-temperature conditions. Wang et al. [13] utilized numerical simulations to investigate the tensile damage and the formation of blast craters in brittle rock due to underground explosions. Li et al. [14] developed a simplified two-dimensional constitutive model for rocks and successfully implemented it into the finite element method to address issues related to rock in engineering applications. Huang et al. [15] also studied the dynamic mechanical properties and fractal characteristics of sandstone under the action of acid solution and dry-wet cycling.

Although existing literature has provided research findings on the impact of chemical corrosion and wet-dry cycles on rocks, systematic research on the coupled effects of these two factors is still lacking. Therefore, further research is needed to study the physical and mechanical properties of rock mass under the combined action of chemical corrosion and wet-dry cycles, in order to provide more in-depth theoretical support for the safety assessment and protective measures of rock engineering. The application of energy theory is pervasive across various aspects of engineering, playing a significant role in each domain [16, 17]. Characterizing the deformation and failure process of rocks from the perspective of energy evolution has become a hot topic in the field of rock mechanics [18–22]. Wang et al. [23, 24] conducted CO_2_ fracturing experiments to investigate the characteristics of rock damage and the distribution and attenuation characteristics of energy in coal-like materials during the CO_2_ fracturing process. Huang et al. [25] established an energy dissipation model for argillaceous siltstone under the action of dry-wet cycling. Chen et al. [26] studied the evolution of total energy and elastic strain energy of sandstone under chemical corrosion. Wang et al. [27] analyzed the impact of strain rate on dissipated energy in soft rock under dynamic impact compression tests. Zha et al. [28] established a damage model for argillaceous siltstone based on the evolution of dissipated energy. Wang et al. [29] investigated the energy dissipation characteristics of rock samples under the action of impact loads. Li et al. [14] developed a two-dimensional rock constitutive model that incorporates strain-dependent elastic modulus, effectively enabling the analysis of mechanical properties of rocks. Investigating the mechanical properties of rocks from the perspective of energy evolution not only reveals the accumulation, transformation, and dissipation of internal energy within rocks under external forces, but also provides a comprehensive understanding of the continuous evolution from elastic deformation to plastic damage, and ultimately to failure. This research approach offers a scientific basis for predicting the stability of rocks. Building upon these findings, this study will further explore the energy evolution mechanism of marble under the coupled effects of chemical corrosion and wet-dry cycling. By conducting loading tests on marble subjected to various chemical solutions and numbers of wet-dry cycles, we obtained the corresponding mechanical parameters and stress-strain curves. Utilizing energy theory, we analyzed the energy dissipation of marble from three dimensions: total energy, elastic strain energy, and dissipated energy. This research provides a novel perspective for the stability assessment and disaster prevention of rock engineering.

## Experimental materials and methods

### Mass loss rate of rock types under acid corrosion

**Fig 1:**
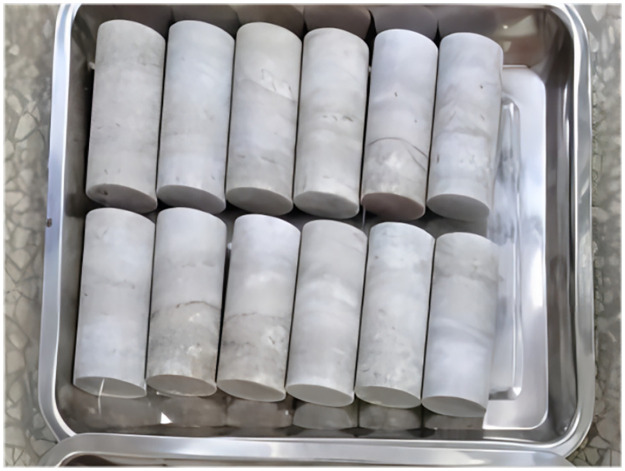
Marble specimens.

The rock samples used in the experiments were collected from Yunnan Province, China, where marble is widely distributed. To ensure the accuracy of the experimental results and minimize sampling errors, all rock samples were taken from the same rock block. In accordance with the testing standards of the International Society for Rock Mechanics, the specimens were machined into standard cylindrical shapes with a height of 100 mm and a diameter of 50 mm. As shown in Fig 1, the ends and sides of the marble specimens were finely ground to ensure that the parallelism of the two end faces and the perpendicularity of the end faces to the sides were all less than 0.02 mm, thereby effectively reducing errors and enhancing the accuracy of the test.

### Chemical solution preparation and wet-dry cycling design

Considering the acidic, neutral, and alkaline characteristics of chemically corrosive environments, the experiment established solutions with pH values of 4, 7, and 10. The acidic solution was prepared by mixing sulfuric acid with distilled water in a specific ratio, the neutral solution was pure distilled water, and the alkaline solution was made by mixing sodium hydroxide with distilled water in a defined ratio. Prior to the experiment, the shaped marble specimens were dried in an oven at 105°C for 24 hours, then removed and allowed to cool to room temperature in a desiccator.

To simulate the effects of wet-dry cycling on marble specimens, a free immersion method was employed. At the commencement of the experiment, the specimens were placed in a water tank, with the initial water level set at one-quarter of the specimen’s height. Subsequently, the water level was incrementally raised to one-half and three-quarters of the specimen’s height every two hours, thereby increasing the contact area between the specimen and water. This procedure was designed to more comprehensively mimic the varying humidity conditions that rocks may experience during natural wet-dry cycling. The entire wet-dry cycling process was sustained for 48 hours, ensuring that the specimens underwent a complete cycle from dry to moist to fully submerged, thereby providing an in-depth understanding of the impact of wet-dry cycling on the durability of marble.

To ensure the stability of the pH value in the chemical corrosion experiment, a strategy of regular monitoring and calibration was implemented. During the wet-dry cycling experiment, the pH value of the solution was monitored in real-time using a pH test pen. Once the specimens were fully submerged in the solution, the pH was measured every four hours with the test pen to ensure the accuracy of the readings. Should the measurements indicate a deviation from the preset pH range, adjustments were made by adding appropriate amounts of sulfuric acid, distilled water, or sodium hydroxide to maintain a constant pH value of the solution. The experiment divided the specimens into 10 groups, comprising one group in a dry state and nine groups subjected to 5, 10, and 20 wet-dry cycling under pH conditions of 4, 7, and 10, respectively, with each group consisting of three specimens. After completing the specified number of cycles, the specimens were tested under uniaxial compression in a naturally air-dried state to assess the mechanical response of marble under various conditions.

### Experimental methods

**Fig 2:**
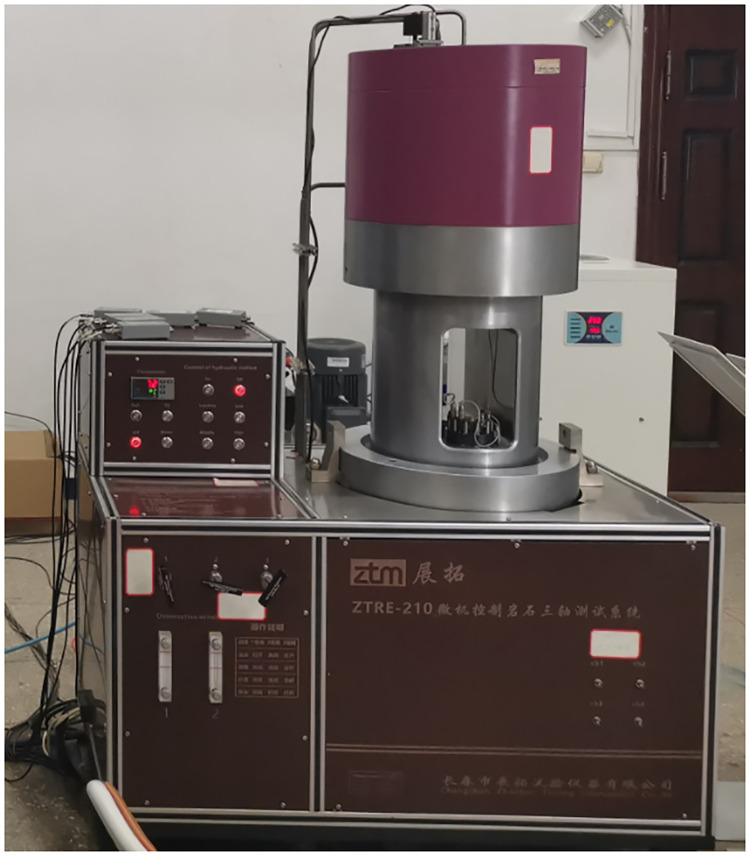
ZTRE-210 Microcomputer Control Rock Triaxial Test System.

In our study, no special permissions were required to access the research site. This is because the experimental site is a public area, open to all, and there are no restrictions on entry. We assure you that our research activities are in full compliance with local laws and regulations, and will not have any adverse impact on the environment or the local community. ZTRE-210 Microcomputer Control Rock Triaxial Test System was selected to perform uniaxial compression loading tests on marble specimens using a displacement control method, with a loading rate set at 0.06mm/min, as shown in Fig 2. To accurately measure the mass changes of marble specimens during the wet-dry cycling process, a high-precision balance is employed for weighing, which allows for the calculation of the mass loss rate. Based on the mechanical parameters and mass data obtained from the experiment, further in-depth analysis of the influence of chemical corrosion and wet-dry cycling on the physicomechanical properties of marble can be conducted.

Based on the indoor test results, the test data were collated to obtain the mass loss rate, average peak strength, average peak strain and average elastic modulus at different stages after corrosion with different acid solutions.

## Experimental results and analyses

### Mass loss rate

To analyze the patterns of mass change of specimens during each stage of wet-dry cycling in chemical solutions of different pH values, the mass loss rate is calculated using the following formula:

$$D=\frac{m_{0}-m_{n}}{m_{0}}\times 100\%$$ (1)

**Fig 3:**
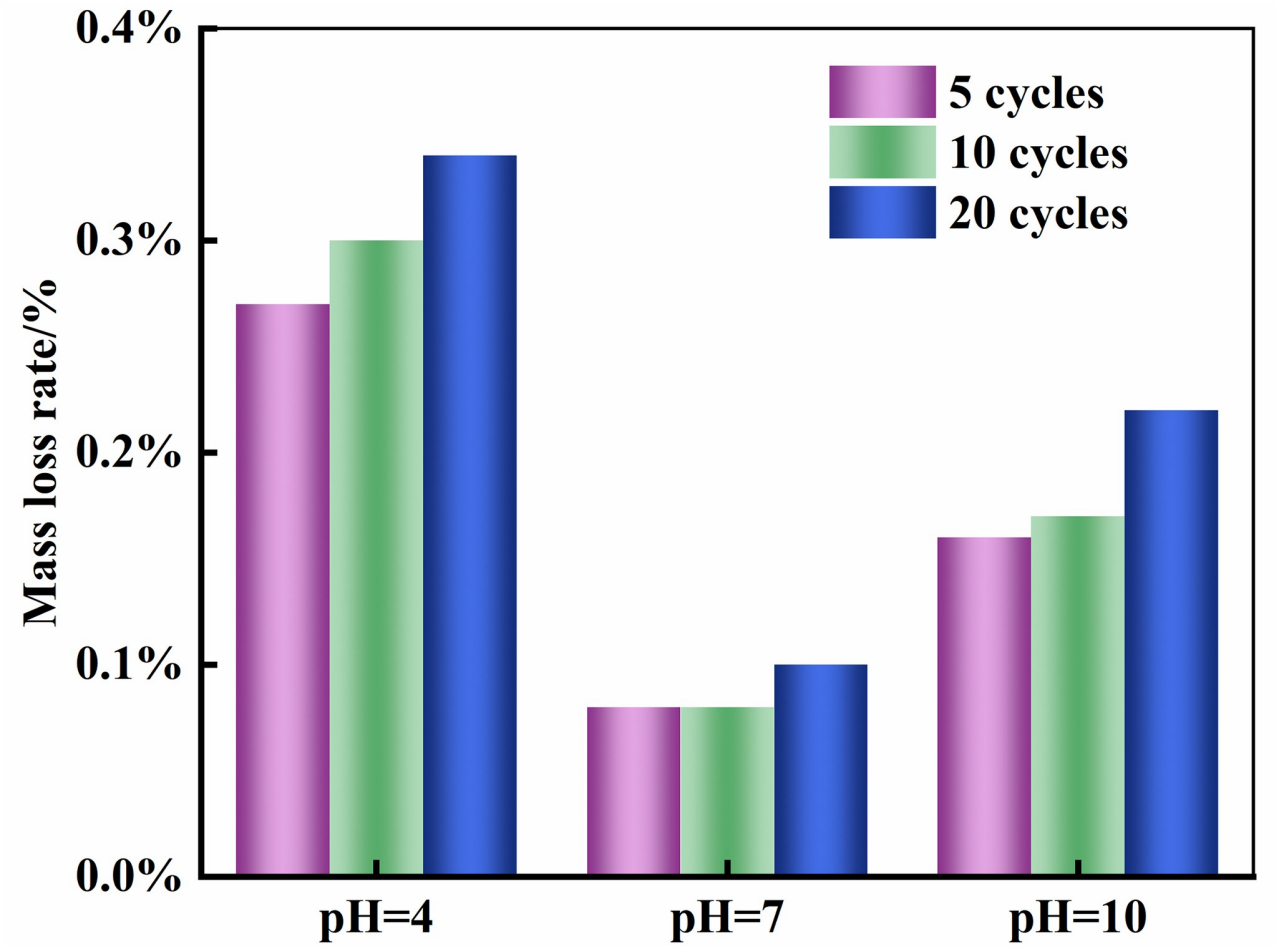
Mass damage rate under different pH values and dry-wet cycles.

Where *m*_0_ represents the mass of the specimen in its initial dry state, and *m*_*n*_ represents the oven-dried mass of the specimen after n cycles of dry-wet exposure. After completing the dry-wet cycles of each stage, the oven-dried mass and the corresponding mass loss rate of the specimens treated with chemical solutions of different pH values are summarized in Table 1. Rock samples subjected to 20 cycles of wet-dry aging were selected as the objects of study, and their masses were measured after 5, 10, and 20 cycles, respectively. The table detailed the mass changes of the specimens after different numbers of wet-dry cycles in various chemical environments. The trend of mass loss rate variation is shown in Fig 3.

**Table 1:** Mass loss rate of marble under different chemical environments and dry-wet cycle conditions.

Cycles/n	Acidic Environment (pH = 4)	Neutral Environment (pH = 7)	Alkaline Environment (pH = 10)	Acidic Environment (pH = 4)	Neutral Environment (pH = 7)	Alkaline Environment (pH = 10)
	m_n_/g	D_n_/%	m_n_/g	m_n_/g	D_n_/%	m_n_/g
0	557.01	0.00	552.95	0.00	554.90	0.00
5	555.50	0.27	552.50	0.08	554.01	0.16
10	555.35	0.30	552.51	0.08	553.95	0.17
20	555.10	0.34	552.40	0.10	553.70	0.22

According to the analysis results depicted in Fig 3, it can be observed that the mass loss rate of the specimens exhibits an increasing trend with the increase in the number of dry-wet cycles. The impact of chemical solutions with different pH values on the mass loss rate of the specimens shows significant differences. Under the same conditions of dry-wet cycling, the mass loss rate of specimens in an acidic solution with a pH value of 4 is significantly higher than that in a neutral solution with a pH value of 7 and an alkaline solution with a pH value of 10. This indicates that acidic and alkaline solutions exert a significant corrosive effect on the specimens. Furthermore, the mass loss rate accounts for a larger proportion in the early stages of dry-wet cycling. In solutions with pH values of 4, 7, and 10, the mass loss rate during the initial stage of dry-wet cycling accounts for 60.53%, 54.55%, and 62.50% respectively, which further confirms that the corrosive impact on rocks is the greatest during the early stages of wet-dry cycling.

### Analysis of mechanical parameters

For the rock samples subjected to various chemical corrosion conditions and the effects of wet-dry cycling, three specimens were selected for uniaxial compression tests each. The resulting mechanical parameters of the rock samples are summarized in Table 2. Within each group of rock samples, a specimen with a compressive strength closest to the group’s average value was selected for in-depth analysis to ensure the representativeness and accuracy of the analysis results. This selection method helps to more precisely evaluate the mechanical behavior of rock samples under different chemical environments and dry-wet cycling conditions.

**Table 2:** Mechanical parameters of marble under different chemical environments and dry-wet cycle conditions.

Cycles/n	Acidic Environment (pH = 4)		Neutral Environment (pH = 7)		Alkaline Environment (pH = 10)	
	Compressive Strength/MPa	Elastic Modulus/GPa	Compressive Strength/MPa	Elastic Modulus/GPa	Compressive Strength/MPa	Elastic Modulus/GPa
0	136.61	19.36	136.61	19.36	136.61	19.36
5	121.54	16.56	133.66	17.98	129.08	18.32
10	111.26	15.48	130.87	17.11	121.90	16.75
20	98.52	12.87	127.86	15.45	117.98	14.83

In this study, a new parameter, the degradation degree *S*_*n*_, is defined to quantify the reduction in mechanical parameters of the specimens after undergoing different numbers of dry-wet cycles n in chemical solutions. The degradation degree can reflect the extent of deterioration of the specimens after experiencing dry-wet cycling, and it is defined as follows:

$$S_{n}=\frac{T_{0}-T_{n}}{T_{0}}\times 100\%$$ (2)

**Fig 4:**
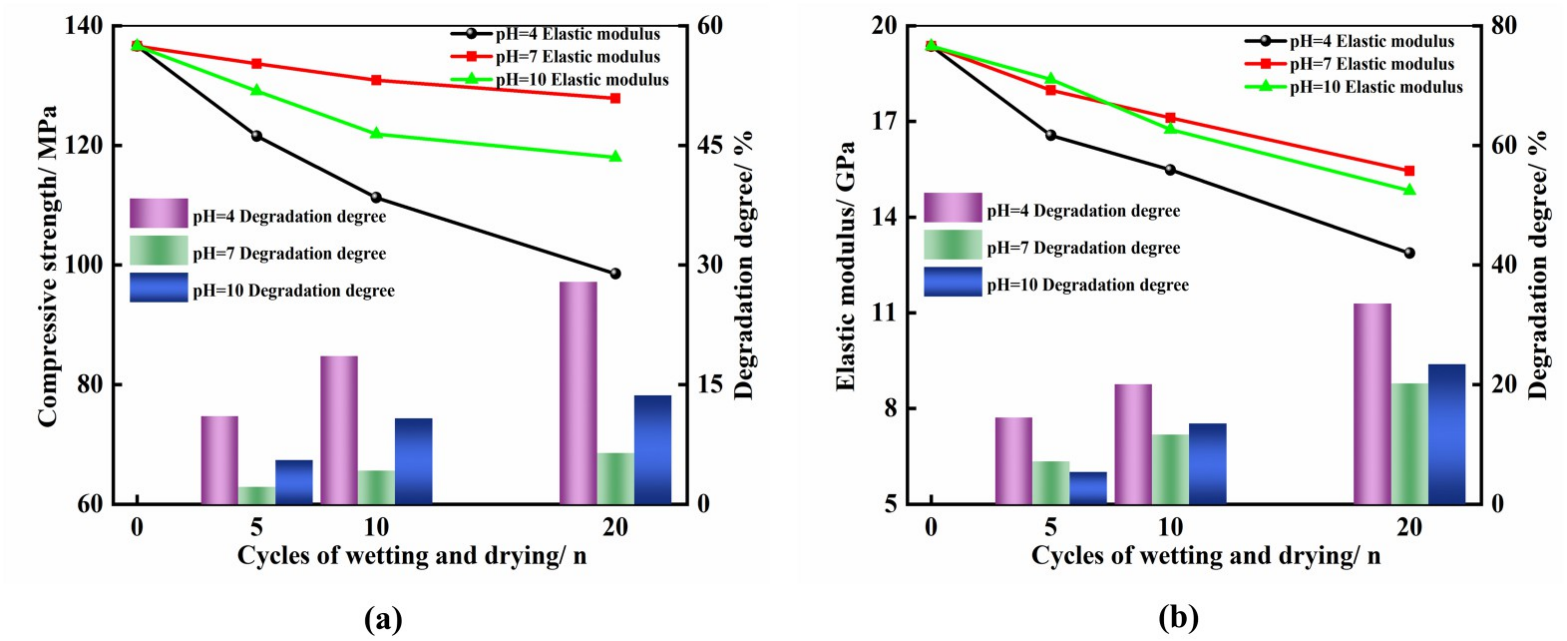
Mechanical parameter trends under different chemical environments and wet-dry cycles. (a) Strength trend chart; (b) Elastic modulus trend chart.

Where *T*_0_ represents the initial mechanical parameter value of the specimen before the dry-wet cycling, which mainly includes parameters such as compressive strength and elastic modulus; *T*_*n*_ represents the mechanical parameter value of the specimen after undergoing *n* cycles of dry-wet exposure, with n taking values of 5, 10, and 20, respectively. This study further explores the impact of different numbers of dry-wet cycles on the degradation degree of uniaxial compressive strength and elastic modulus under acidic, neutral, and alkaline environments, and the trend of these changes is visually presented in Fig 4.

**Fig 5:**
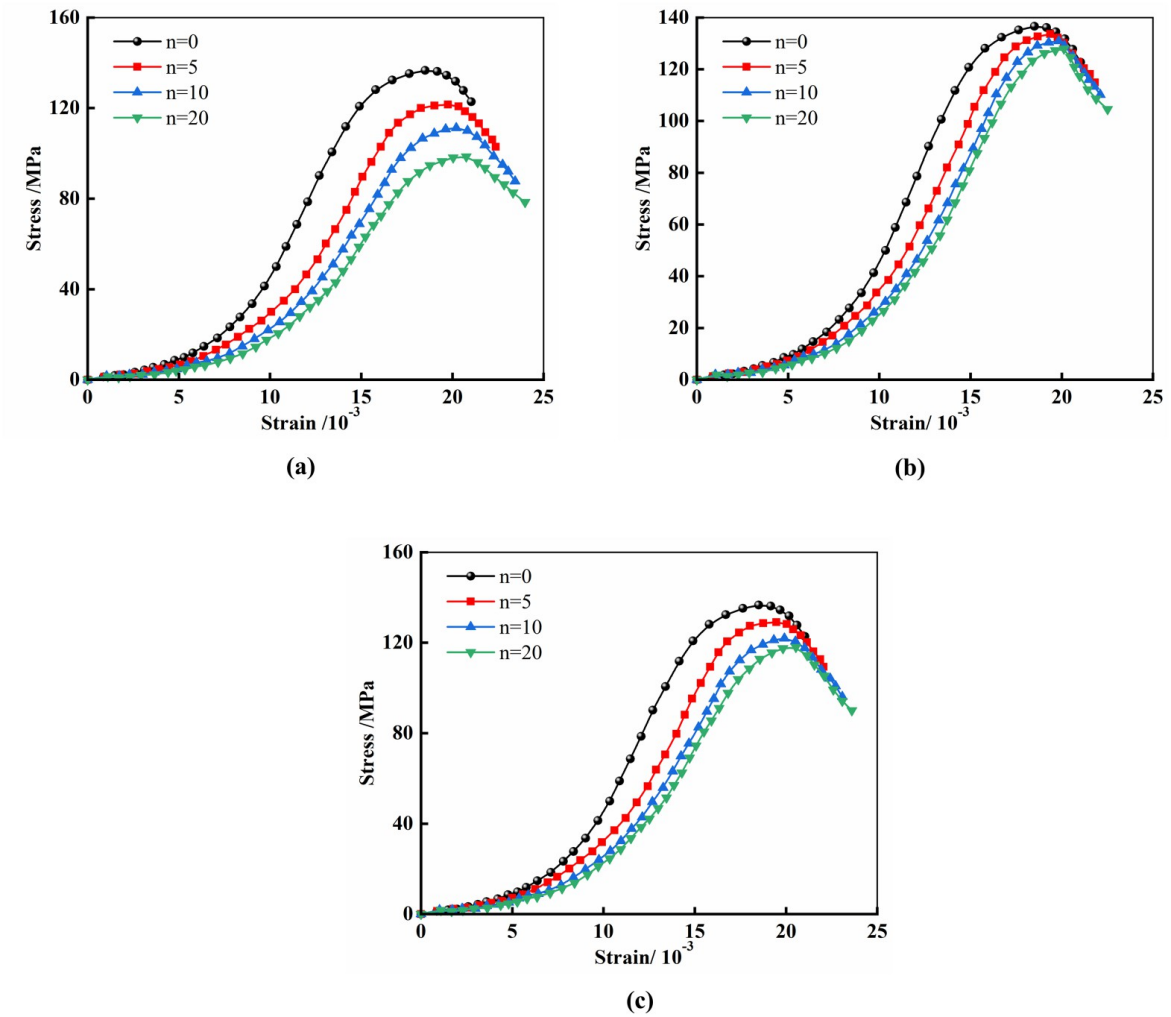
Stress-strain curves. (a) pH = 4; (b) pH = 7; (c) pH = 10.

According to the analysis results depicted in Fig 4, it can be observed that in solutions with three different pH values, the uniaxial compressive strength shows a decreasing trend with the increase in the number of dry-wet cycles. Under the same conditions of dry-wet cycling, the acidic solution environment has the most significant impact on the degradation of the specimens. For instance, after the 5th cycle of dry-wet exposure, the compressive strength of specimens in acidic and alkaline solutions decreased by 11.03% and 5.51%, respectively, while the decrease in the neutral environment was the smallest, at 2.16%. Furthermore, after the 20th cycle of dry-wet exposure, the compressive strength of specimens in the acidic solution dropped to 98.52 MPa, with a reduction of 27.88%; in the alkaline solution, it dropped to 117.98 MPa, with a reduction of 13.64%; and in the neutral solution, it dropped to 127.86 MPa, with a reduction of 6.41%. Additionally, regardless of whether in acidic, neutral, or alkaline environments, as the number of dry-wet cycles increases, the degradation effect on the uniaxial compressive strength of the specimens shows a decreasing trend, indicating that the degradation effect in the early stages of dry-wet cycling is significantly greater than in the later stages. The trend of the degradation degree of the elastic modulus is similar to that of the uniaxial compressive strength, but under the same conditions of dry-wet cycling, the degradation effect on the elastic modulus is generally more pronounced. In the acidic solution environment, as the number of dry-wet cycles increases, the degradation degree of the elastic modulus is 14.46%, 20.04%, and 33.52%, respectively. In the neutral solution environment, the degradation degrees are 7.13%, 11.62%, and 20.20%, respectively. In the alkaline solution environment, the degradation degrees are 5.37%, 13.48%, and 23.40%, respectively. It is evident that under the same number of wet-dry cycles, the degradation of the elastic modulus in an acidic environment is significantly more pronounced than in alkaline and neutral environments. However, the degree of degradation in neutral and alkaline environments does not differ significantly. Furthermore, the rate of degradation of the elastic modulus is relatively rapid during the initial stages of wet-dry cycling, and this rate gradually decelerates as the cycling progresses. This is attributed to the fact that in acidic environments, rocks are more susceptible to chemical reactions with acids, which disrupt their mineral composition and deteriorate their structure. The typical stress-strain curves of marble specimens under dry-wet cycling in environments with different pH values are shown in Fig 5.

The analysis of the test results indicates that the uniaxial compressive stress-strain curves of specimens corroded in chemical environments with different pH values exhibit similar phased characteristics compared to those under natural conditions, including the compaction phase, elastic phase, and yield phase. Specifically, during the compaction phase, the stress-strain curve of the specimens under natural conditions shows a relatively short concave segment, while the specimens corroded by acidic and alkaline environments exhibit a more pronounced concave segment, and the slope of the stress-strain curve decreases after chemical solution corrosion. Particularly, specimens subjected to wet-dry cycling in an environment with a pH value of 7 maintained a high degree of consistency in their stress-strain curves compared to those under natural conditions. These observations reveal the impact of the chemical environment on the mechanical behavior of rock materials, especially in terms of the stress-strain response during the compaction and elastic phases. Additionally, the impact of the pH value of the chemical environment on the mechanical properties of the specimens indicates that a neutral environment has a lesser effect on the mechanical properties of marble compared to acidic and alkaline environments.

## Analysis of energy evolution in marble

### Principles of energy calculation

The process of sample deformation and rupture fundamentally involves the accumulation, dissipation, and release of energy. When studying the deformation behavior of rock samples under external forces, it is assumed that during the loading process, there is no heat exchange between the specimen and the external environment, meaning the specimen is in an adiabatic closed system. Under this condition, the work done by the external force on the specimen is transformed into the total input energy of the system. According to the first law of thermodynamics, which is the law of conservation of energy, the change in internal energy of the system is equal to the amount of work done on the system, which can be expressed as:

$$U=U^{d}+U^{e}$$ (3)

**Fig 6:**
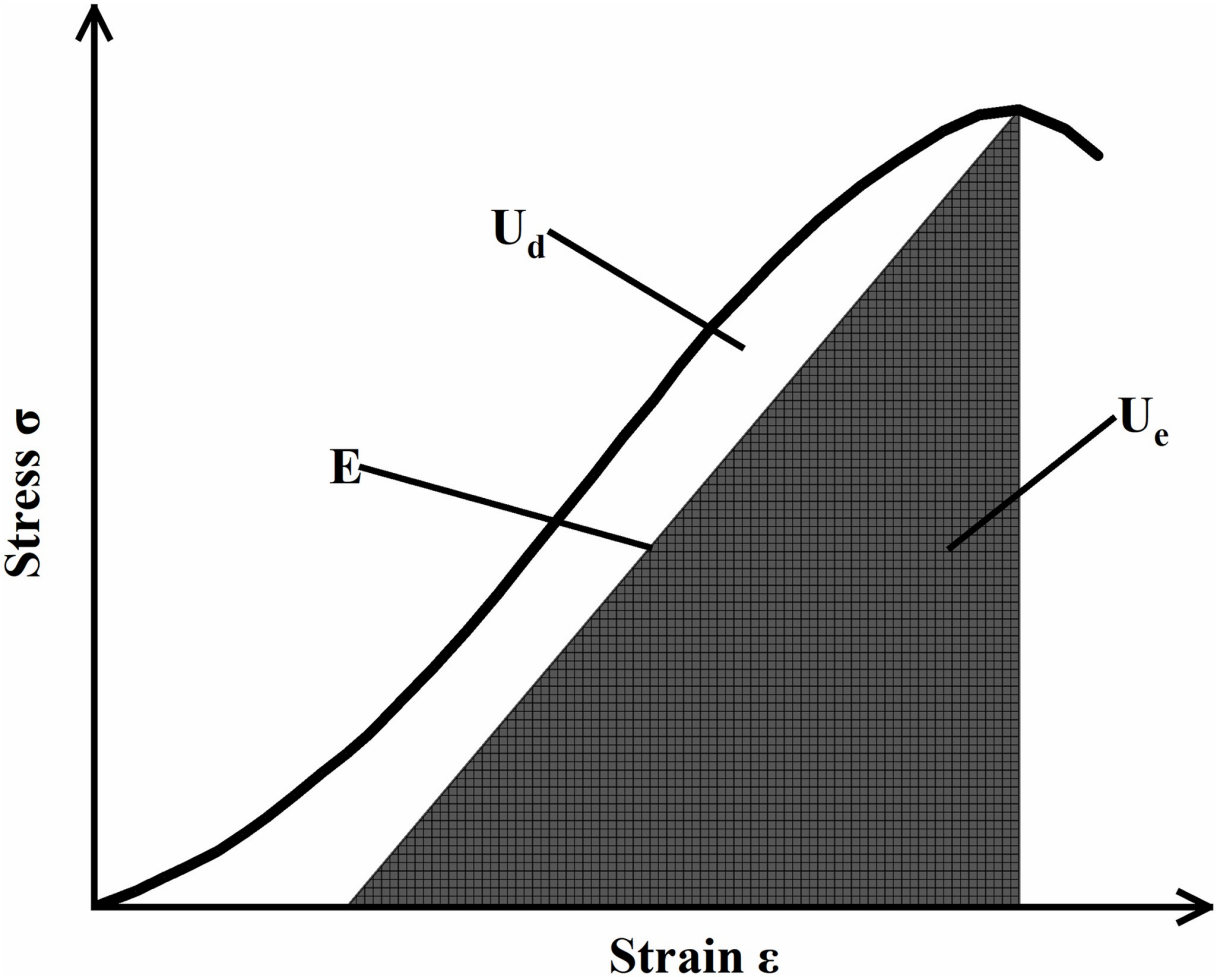
Relationship between dissipation energy and elastic strain energy.

In the equation: *U*^*d*^ represents the dissipated energy, and *U*^*e*^ represents the elastic strain energy. The stress-strain relationship curve of the marble specimen and the relationship between dissipated energy and releasable elastic strain energy are shown in Fig 6.

The energy of each part of the rock unit in the principal stress space is represented as:

$$U^{e}=\frac{1}{2}\sigma_{1}\varepsilon_{1}^{e}+\frac{1}{2}\sigma_{2}\varepsilon_{2}^{e}+\frac{1}{2}\sigma_{3}\varepsilon_{3}^{e}$$ (4)

In the equation: *σ*_1_, *σ*_2_ and *σ*_3_ represent the stresses in the three loading directions; $\varepsilon_{1}^{e}$, $\varepsilon_{2}^{e}$ and $\varepsilon_{3}^{e}$ represent the elastic strains corresponding to the stresses. According to Hooke’s Law:

$$U^{e}=\frac{1}{2E}\left[\sigma_{1}^{2}+\sigma_{2}^{2}+\sigma_{3}^{2}-2\nu \left(\sigma_{1}\sigma_{2}+\sigma_{2}\sigma_{3}+\sigma_{1}\sigma_{3}\right)\right]$$ (5)

By combining the above equations, the damage constitutive model under uniaxial compression can be established. In the equation: *E* is the initial elastic modulus; *ν* is Poisson’s ratio. For uniaxial compression tests, the formula for calculating elastic strain energy can be simplified to [30]:

$$U^{e}=\frac{\sigma_{1}^{2}}{2E}$$ (6)

The total energy calculation formula is given by:

$$U=\int \sigma_{1}d\varepsilon_{1}=\overset{n-1}{\underset{i=1}{\sum }}\int_{\varepsilon_{i}}^{\varepsilon_{i+1}}\sigma_{i}d\varepsilon_{1}=\overset{n-1}{\underset{i=1}{\sum }}\frac{\varepsilon_{i+1}-\varepsilon_{i}}{2}\left(\sigma_{i+1}+\sigma_{i}\right)$$ (7)

In the equation: *σ*_*i*_ and *ε*_*i*_ represent the stress and strain values at each point on the stress-strain curve of the rock specimen, respectively. Therefore, the dissipated energy during the deformation and failure process of the rock specimen is obtained as:

$$U^{d}=U-U^{e}$$ (8)

### Laws of energy evolution

**Fig 7:**
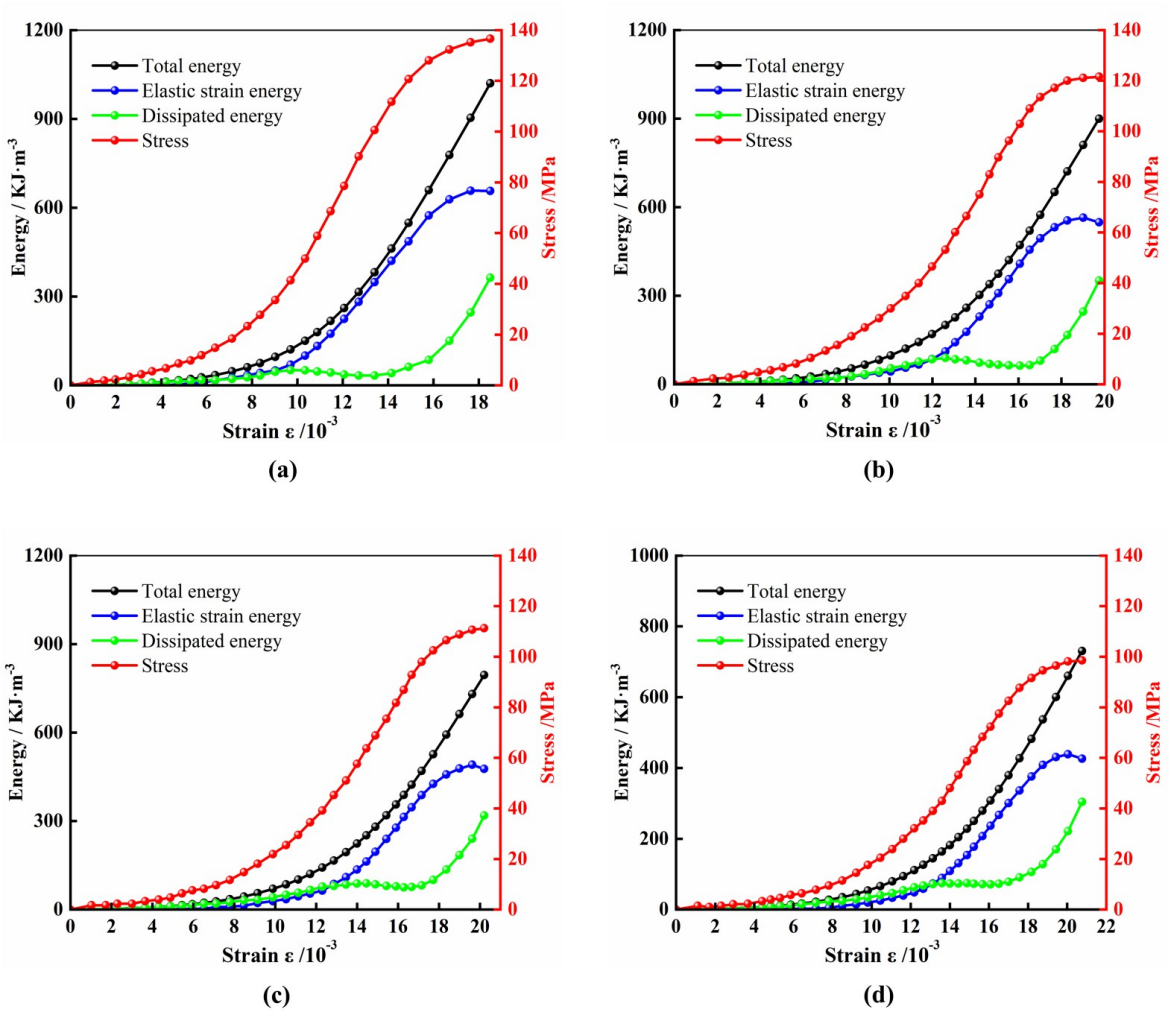
Patterns of energy changes in marble samples and their stress-strain curves under dry-wet cycles. (a) 0 wet-dry cycles; (b) 5 wet-dry cycles; (c) 10 wet-dry cycles; (d) 20 wet-dry cycles.

Studying the patterns of energy change in marble during the loading process, as well as the intrinsic connections between these changes and the strength and failure of marble, is of significant importance for understanding the strength variation characteristics and failure mechanisms of marble under load. Using the aforementioned formulas, it is possible to calculate the total energy, elastic strain energy, and dissipated energy of specimens during deformation and failure under different chemical corrosion conditions. Since marble samples are of a brittle type with an inconspicuous post-peak stage, this study selects the stage before the marble samples reach the peak stress for energy calculation and analysis. Considering the significant degradation of marble under acidic conditions in dry-wet cycling, the analysis focuses on marble samples subjected to different dry-wet cycles in a pH 4 acidic solution. The energy change patterns of marble samples after different dry-wet cycles and their stress-strain curves are shown in Fig 7.

During the compaction phase of the rock, the growth rates of total energy, elastic strain energy, and dissipated energy are relatively slow. In this process, the micro-fractures within the specimen gradually close, and the frictional closure of these micro-fractures causes the energy absorbed by the rock sample to be mainly transformed into dissipated energy. In the later stages of the compaction phase, the growth rate of the dissipated energy in the rock samples under natural conditions begins to slow down. However, in rock samples cured with acid solution, the growth rate of the dissipated energy does not show a decreasing trend, and the more times of dry-wet cycles, the more significant this phenomenon becomes.

In the elastic phase, the rock sample mainly accumulates elastic strain energy. In the early stages of this phase, the growth rates of the total energy and elastic strain energy in the rock samples under natural conditions are almost the same, and the curves of the two are highly coincident, indicating that the total energy is almost entirely composed of elastic strain energy, while the dissipated energy remains at a very low level. However, due to the impact of acid corrosion, the total energy and elastic strain energy curves of the rock samples with different numbers of dry-wet cycles begin to separate, and the degree of separation increases with the increase in the number of dry-wet cycles, and the dissipated energy continues to grow at a certain rate in this stage. The reason is that after the compaction phase, the internal structure of the rock sample under natural conditions is improved, and at this time, the total energy is almost entirely composed of elastic strain energy. Rock samples corroded by acidic solutions develop a higher density of micro-defects, leading to more severe structural deterioration. Consequently, the dissipative energy associated with friction, sliding, and the generation of new fractures within the fissures is also significantly increased.

In the plastic phase, the energy evolution trend of the rock samples is basically similar under both natural conditions and acid solution curing conditions. In this stage, the internal fractures develop and pass through rapidly, the irreversible plastic deformation increases, leading to a rapid increase in dissipated energy, while the elastic strain energy grows slowly. When approaching failure, the fractures develop to form a rupture surface, the elastic strain energy reaches the limit of energy storage, the corresponding dissipated energy increases sharply, and then the rock sample undergoes overall failure.

### Evolution of peak energy under chemical corrosion-dry-wet cycle couple

**Fig 8:**
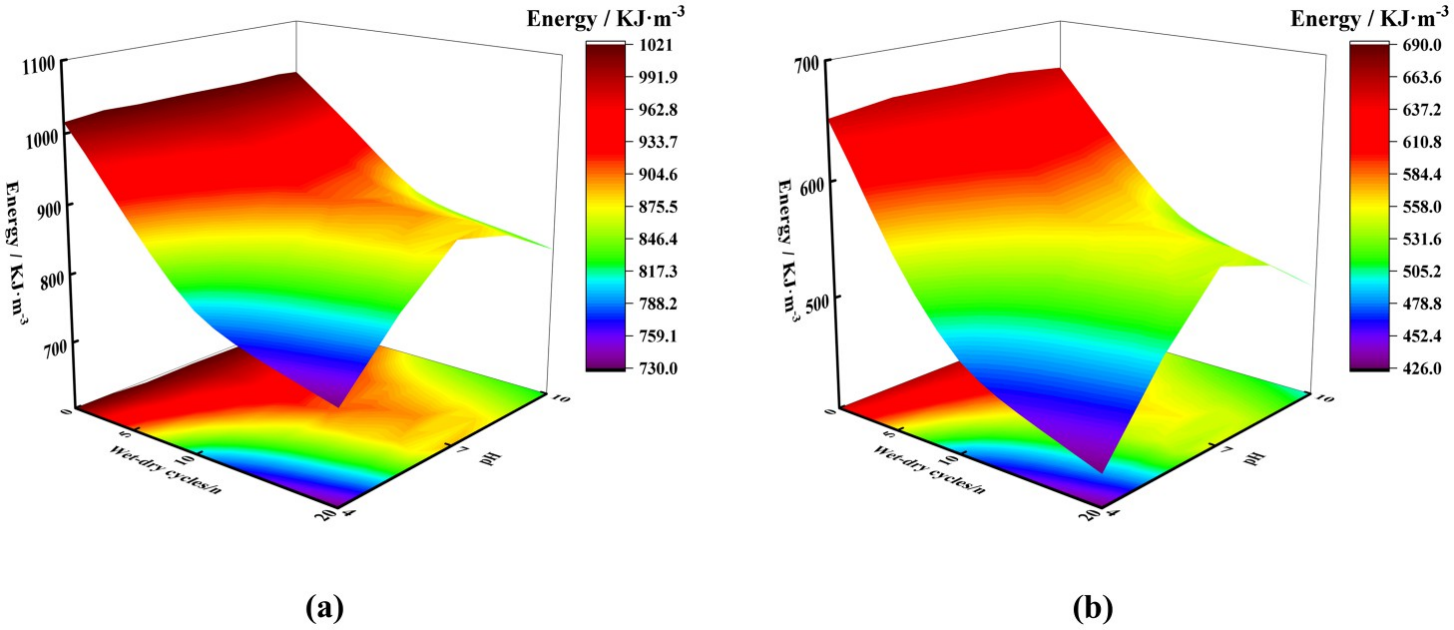
Total energy and elastic strain energy under different conditions. (a) Total energy; (b) Elastic strain energy.

According to the observations from Fig 8, the peak total energy of marble at the point of failure is jointly influenced by the number of dry-wet cycles and the pH value of the corrosive solution. As the number of dry-wet cycles increases, the total energy shows a declining trend. Under the same conditions of dry-wet cycling, the reduction in total energy is most significant in acidic environments, followed by alkaline environments, with the smallest decrease in neutral environments. Additionally, the trend of change in elastic strain energy is consistent with that of the total energy.

The peak value of elastic strain energy represents the limit of the rock sample’s energy storage capacity, indicating the amount of energy that can be stored within the sample. The corrosive action of chemical solutions has a significant negative impact on the internal energy storage capacity of the rock samples. The main reason for this phenomenon is that chemical corrosion leads to the formation of more micro-fractures within the samples. Under the same strain conditions, rock samples in their natural state primarily store elastic strain energy through deformation. In contrast, rock samples that have undergone acidic chemical corrosion have a reduced energy storage capacity due to increased porosity and the concurrent occurrence of plastic deformation.

**Fig 9:**
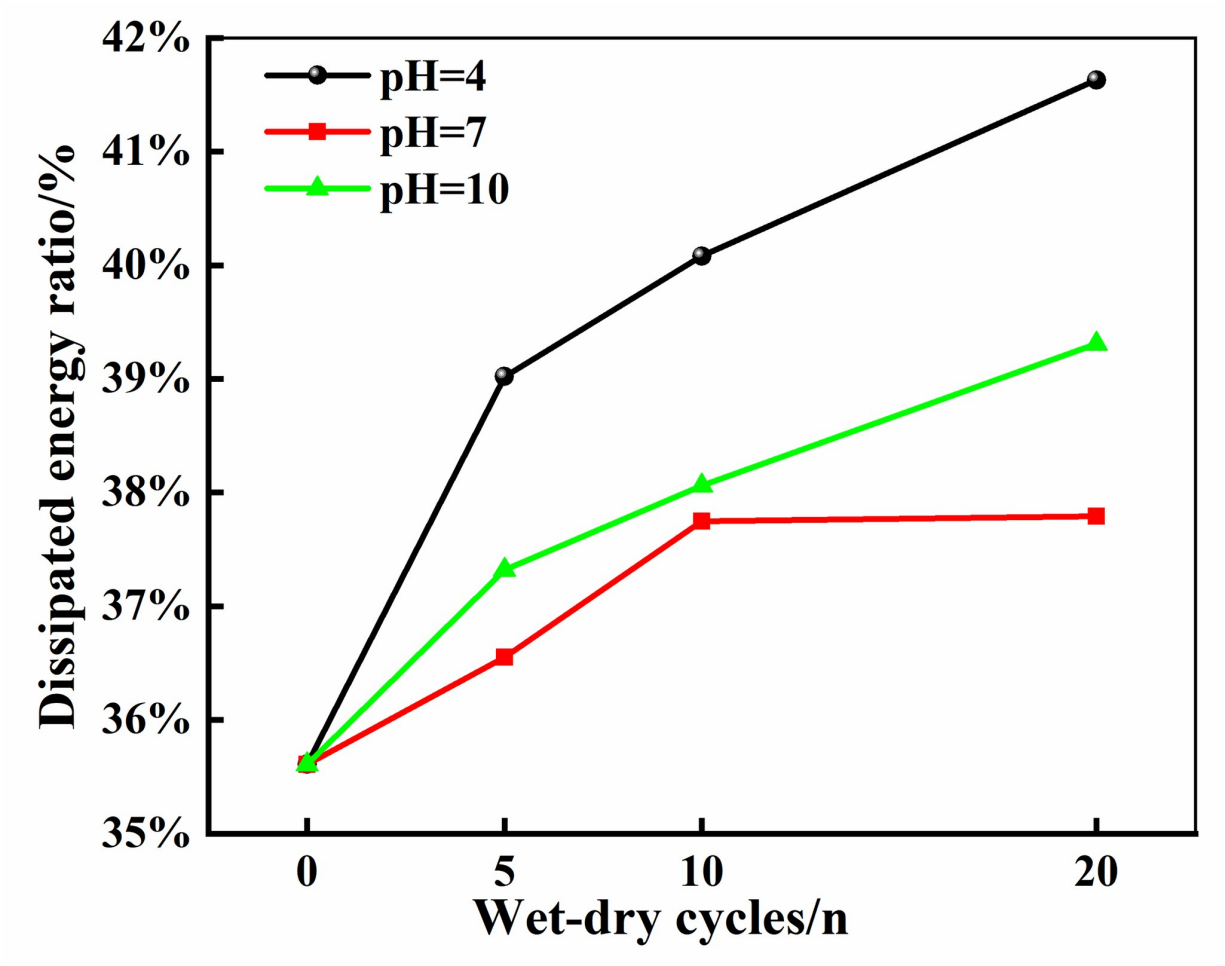
Dissipated energy ratio.

To further explore the impact of dry-wet cycling on the performance of marble, this study analyzed the ratio of dissipated energy to total energy at the point of sample failure, as shown in Fig 9. As the number of dry-wet cycles increases, the degree of chemical damage intensifies, leading to a significant rise in the proportion of dissipated energy. This trend reflects that with increasing chemical damage, a larger proportion of the energy absorbed from the external environment by marble is transformed into dissipated energy, used for the rock’s fracture and deformation. Correspondingly, this indicates that the capacity for plastic deformation in marble is also enhanced.

## Conclusions

By investigating the mechanical characteristics of rocks under various chemical environments and wet-dry cycling conditions, as well as the application of energy dissipation principles under these conditions, our study reveals the stress-strain response and energy dissipation mechanisms of rocks under such conditions. This provides a foundation for the performance assessment of rock materials in complex environments and offers a basis for the implementation of underground engineering projects. The main conclusions are as follows:

With the increment of wet-dry cycle counts, the mass loss rate of specimens escalates progressively. It is observed that chemical solutions with varying pH values exert a significant influence on this rate, with acidic solutions (pH = 4) leading to the most pronounced mass loss. In contrast, the impact of alkaline (pH = 10) and neutral (pH = 7) solutions is considerably less. Notably, the mass loss during the initial stages of wet-dry cycling constitutes a substantial fraction of the overall loss, highlighting the critical role of early-stage corrosion.

Uniaxial compression tests indicate that the compressive strength and elastic modulus of marble specimens are subject to varying degrees of deterioration under the synergistic action of wet-dry cycles and chemical corrosion. Especially in the acidic environment, the degree of degradation of the elastic modulus is significantly higher than in neutral and alkaline environments. In addition, the rate of deterioration in the early stages of dry-wet cycling is faster, and it gradually slows down later.

During the consolidation phase, the closure of internal micro-fractures in rock samples results in an increase in dissipated energy. As the samples transition into the elastic phase, the acid corrosion leads to a decoupling of total energy from elastic strain energy, with dissipated energy continuing to rise, indicative of structural degradation and a proliferation of micro-defects. Within the plastic phase, rock samples display analogous trends in energy evolution under both natural conditions and acidic environments, characterized by a precipitous increase in dissipated energy, culminating in the comprehensive rupture of the rock samples.

The total energy diminishes incrementally with the proliferation of wet-dry cycles, with the acidic environment exerting the most pronounced reduction in total energy, followed by the alkaline environment, while the neutral environment demonstrates the minimal impact. The trajectory of elastic strain energy mirrors the overall energy descent, signifying that chemical corrosion substantially impairs the rock sample’s capacity to retain energy, predominantly due to the proliferation of micro-fractures instigated by chemical weathering. Subsequent analyses reveal that as the wet-dry cycling intensifies, there is a notable upsurge in the proportion of dissipated energy, concurrent with an escalation in the propensity for plastic deformation.
